# Molecular Insights Into *O*-Linked Glycan Utilization by Gut Microbes

**DOI:** 10.3389/fmicb.2020.591568

**Published:** 2020-11-05

**Authors:** Kevin J. González-Morelo, Marco Vega-Sagardía, Daniel Garrido

**Affiliations:** Department of Chemical and Bioprocess Engineering, School of Engineering, Pontificia Universidad Católica de Chile, Santiago, Chile

**Keywords:** glycans, prebiotics, GMP, mucin, microbiota, glycoprotein

## Abstract

*O*-linked glycosylation is a post-translational modification found mainly in eukaryotic cells, which covalently attaches oligosaccharides to secreted proteins in certain threonine or serine residues. Most of *O*-glycans have *N*-acetylgalactosamine (GalNAc) as a common core. Several glycoproteins, such as mucins (MUCs), immunoglobulins, and caseins are examples of *O*-glycosylated structures. These glycans are further elongated with other monosaccharides and sulfate groups. Some of them could be found in dairy foods, while others are produced endogenously, in both cases interacting with the gut microbiota. Interestingly, certain gut microbes can access, release, and consume *O*-linked glycans as a carbon source. Among these, *Akkermansia muciniphila*, *Bifidobacterium bifidum*, and *Bacteroides thetaiotaomicron* are prominent *O*-linked glycan utilizers. Their consumption strategies include specialized *α*-fucosidases and α-sialidases, in addition to endo-*α*-*N*-acetylgalactosaminidases that release galacto-*N*-biose (GNB) from peptides backbones. *O*-linked glycan utilization by certain gut microbes represents an important niche that allows them to predominate and modulate host responses such as inflammation. Here, we focus on the distinct molecular mechanisms of consumption of *O*-linked GalNAc glycans by prominent gut microbes, especially from mucin and casein glycomacropeptide (GMP), highlighting the potential of these structures as emerging prebiotics.

## Introduction

There is a great interest regarding the impact and modulation of the gut microbiota through our diet. Among several dietary interventions, consumption of fibers and prebiotics has mainly been considered as positive for our health (Sawicki et al., 2017). A recent definition of prebiotic corresponds to a substrate that is selectively utilized by host microorganisms conferring a health benefit (Gibson et al., 2017). These benefits include a reduced microbial load of pathogens (Gibson et al., 2005), stimulation of the immune system (Shokryazdan et al., 2017), and lower allergy rates (Brosseau et al., 2019). These effects are attributed in part to the ability of prebiotics to be fermented by healthy microorganisms and stimulate the production of certain short-chain fatty acids (SCFA), especially butyrate (Rivière et al., 2016).

Traditionally, carbohydrates such as inulin and fructo-oligosaccharides (FOS) have been studied as prebiotics (Vandeputte et al., 2017; Wilson and Whelan, 2017; BeMiller, 2019). In general, these plant-derived prebiotics have a simple structure containing one monosaccharide and repeats of one linkage. In contrast, host-derived glycans such as human milk oligosaccharides (HMO) and those found in glycoproteins, are structurally more complex and thought to be more suitable and selective toward beneficial members of the gut microbiota. HMO are complex glycans that promote the growth and activity of beneficial gut microbes such as infant-gut associated bifidobacteria, among other effects (Thompson et al., 2018). Several advances have been made to synthesize HMO at the industrial level, and they are currently being added as functional ingredients to infant formula (Puccio et al., 2017).

Besides, some human and bovine milk proteins possess similar glycans to HMO in their structures. Glycosylation is a post-translational modification where oligosaccharides are covalently bound to asparagine (*N*-glycans), or serine or threonine (Ser/Thr; *O*-glycans; Vliegenthart, 2017). These glycans serve as signaling molecules for secretion and other cellular processes, providing increased resistance to proteolysis (Baum and Cobb, 2016). These glycans are being proposed as emerging prebiotics due to their similarity to host-derived glycans compared to plant-derived prebiotics and their enrichment of dominant and health-promoting gut microbes, such as *Bacteroides* spp. (*Ba.*), *Bifidobacterium* spp. (*Bi.*), and *Akkermansia muciniphila* (Bergstrom and Xia, 2013; Kirmiz et al., 2018). Several molecular adaptations for accessing and consuming *N*- and *O*-glycans have been described in gut microorganisms, indicating that the gut microbiota is quite adapted for metabolizing these oligosaccharides. Importantly, some of these bioactive glycans could be also be found in dairy streams, warranting a wide availability for potential prebiotic use for the food industry.

*N*-linked glycans are complex oligosaccharides that possess *N*-Acetylglucosamine (GlcNAc) as a common core, conjugated with an additional GlcNAc and three mannose (Man) residues, forming a Man_3_GlcNAc_2_ motif found in all *N*-glycans. *N*-glycans could be further modified by extensive mannosylation (high-mannose *N*-glycans) or by lactosamine chains (Gal*β*1-4GlcNAc; complex *N*-glycans). Terminal fucose (Fuc) and *N*-acetylneuraminic acid (NeuAc) modifications are commonly added to complex *N*-glycans (Stanley et al., 2017). *N*-glycans are characteristic of immunoglobulins and lactoferrin in milk (Le Parc et al., 2015; Davis et al., 2016). *N*-glycans are an example of host-derived oligosaccharides that can be used by beneficial microbes. *Bifidobacterium longum* subsp. *infantis* ATCC 15697 is a dominant beneficial infant gut microbe that has been shown to access *N*-glycans *in vitro* by a specialized endo-*β*-*N*-acetylglucosaminidase (Garrido et al., 2012b). It also shows vigorous growth *in vitro* using *N*-glycans (Karav et al., 2016). The released *N*-glycans from milk glycoproteins such as lactoferrin and immunoglobulins have been well characterized (Le Parc et al., 2015). These results, while promising, are yet to be tested *in vivo* for claiming any prebiotic effect.

**Figure 1:**
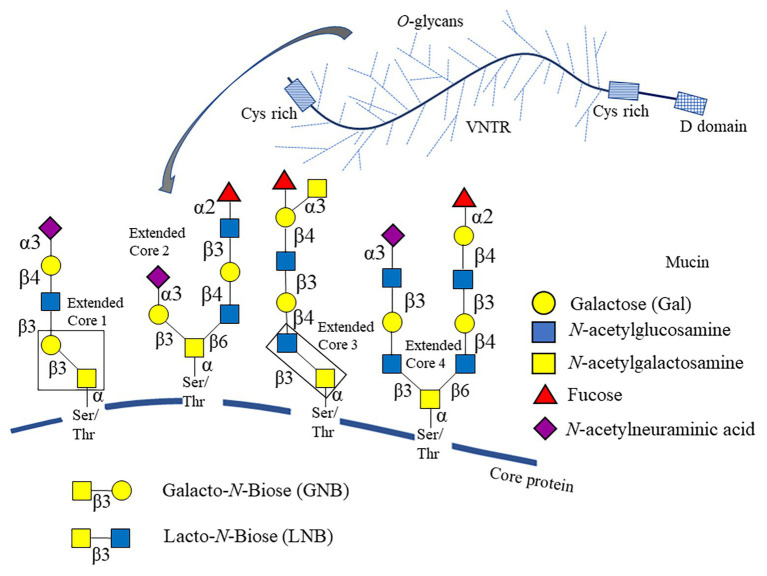
Schematic representation of *O*-glycan cores linked to mucin. The *O*-glycans structures linked to Ser/Thr residues from mucin (MUC) core protein are composed of four dominant extensible cores (Cores 1–4), with the addition of residues such as Gal, *N*-acetylgalactosamine (GlcNAc), fucose (Fuc), and *N*-acetylneuraminic acid (NeuAc). Carbohydrate legends are shown to the right. The distribution of variable number of tandem repeats (VNTR) and other domains are also shown.

Less attention has been paid to *O*-linked glycans. These are characterized by an *N*-acetylgalactosamine (GalNAc) residue linked to Ser/Thr as a common core. GalNAc is usually bound to Gal*β*1-3, forming galacto-*N*-biose (GNB) as a building block in Core 1 and Core 2, which could be further extended forming larger chains similar to *N*-glycans. *O*-linked glycans are a significant component of mucins (MUCs; Figure 1), also found in immunoglobulins and *κ*-caseins (Magnelli et al., 2011). *O*-glycosylated proteins have a higher resistance to proteolysis (Boutrou et al., 2008; Kesimer and Sheehan, 2012), probably reaching lower sections of the gut and interacting the gut microbiota. Therefore, it is not surprising that gut microbes have evolved strategies to utilize *O*-linked glycans as a carbon source.

Prominent gut microbes such as *A. muciniphila*, *Bifidobacterium bifidum*, and *Bacteroides thetaiotaomicron* are representative species capable of *O*-linked glycan consumption. However, the mechanisms involved and the consequences of this process for the host are not fully known. This review aims to summarize and discuss current research regarding the structures of *O*-linked glycans, their potential prebiotic activity, and consumption by gut microorganisms.

## Structures Of *O*-Linked Glycans

The elongation of *O*-linked glycans and their attachment to secreted proteins begins in the Golgi apparatus (Brockhausen and Stanley, 2017). A polypeptide-*N*-acetyl-galactosaminyltransferase (ppGalNAcT) catalyzes the addition of a GalNAc*α*1 to a Ser/Thr available as a glycosylation site (Varki and Lowe, 2009). Several glycosyltransferases act in conjunction to attach sugar residues in single *O*-glycans. The Core 1 *O*-glycan (Gal*β*1-3GalNAcα1-Ser/Thr) is the first synthesized, and then the Core 2 unit [Galβ1-3(GlcNAcβ1,6)GalNAcα1-Ser/Thr; Itano, 2019].

Here, we will focus on two model glycoproteins containing *O*-glycans: MUC and glycomacropeptide (GMP). The wide availability of mucins from animal sources makes them reference glycoproteins for the study of their contribution to host processes and their interaction with the gut microbiota. Similarly, GMP is a glycopeptide derived from cheesemaking, available in large quantities. It contains neutral and acidic *O*-glycans, which are shorter and simpler compared to mucin *O*-glycans. Both types of glycans have been shown to interact with the gut microbiota and could be considered promising prebiotics considering their stimulation of beneficial gut microbes.

### *O*-Linked Glycans From Mucin

Mucins are highly glycosylated (up to 90%), high-molecular-weight (200 kDa–200 MDa), and large (Rg 10–300 nm) extracellular glycoproteins that serve as a dense barrier between the intestinal lumen and epithelium (Kesimer and Sheehan, 2012). They are generally found in epithelial tissues in the gastrointestinal tract and certain secretions. They provide a crucial role in forming a physicochemical barrier against the luminal compartment through their gel-forming properties (Bansil and Turner, 2018). Interestingly, mucin serves as a scaffold for the attachment and colonization by certain microorganisms (Ringot-Destrez et al., 2017). Two different mucus layers have been identified: an outer mucus layer (containing 10^6^ microbial cells/g) and a tight inner mucus layer (10^5^ microbial cells/g; Atuma et al., 2001). Mucins can be found in different parts of the human body: kidneys and bladder, respiratory and urinary tracts, among others (Atuma et al., 2001). They are produced by goblet cells, specialized secretory cells found in the epithelial layer (Lamacchia et al., 2018).

There are at least 20 different MUC genes, whose products are classified into two families: secreted and transmembrane mucins (Corfield, 2015; Dhanisha et al., 2018). Secreted mucins can be small and soluble, or large gel-forming mucins. The latter are able to cross-link, forming complex networks and contributing to the viscosity of mucins. MUC5AC is among the most studied mucin being secreted by epithelial cells in the stomach, while MUC6 is secreted in the deep gastric glands in the stomach and ileum (Magalhães et al., 2016). MUC2 is the most abundant secreted mucin in the small intestine and the colon (Corfield, 2000; Arike et al., 2017). Transmembrane mucins participate primarily in cellular adhesion, while secreted mucins are responsible for viscoelasticity for both the inner and outer mucus layers (Lillehoj et al., 2013; Demouveaux et al., 2018). All these differences between secreted and membrane-bound mucins contribute to the dynamic properties of the mucus layer across the GI tract.

Mucins could also be found in secretions and milk. MUC1 is a transmembrane mucin expressed on the apical surface of most epithelial cells (Ross et al., 2015). This type of mucin can be found in breast milk, which is transferred to the milk fat globule membrane upon its secretion from the cells. MUC15 is also a mucin-type isolated from bovine milk fat globule membranes (Bernard et al., 2018). The function of milk mucins has not been extensively studied, but it is suggested to be mostly structural (Donovan, 2019).

Eight cores of *O*-glycan structures have been identified in mucins, four of them being the most predominant (Core 1–4; Figure 1). These *O*-glycans are found in the region known as the variable number of tandem repeats (VNTR; Arike and Hansson, 2016). The VNTR section is rich in Ser/Thr, which can make up to 80% of the weight of the mucin. In MUC2, VNTRs are accompanied by two cysteine-rich regions at their ends and a D domain involved in mucin polymerization (Arike and Hansson, 2016). Depending on the tissue, MUC5AC and MUC6 could be glycosylated with predominant Core 1–2 structures (Jin et al., 2017). The *O*-linked glycans of colonic MUC2 are predominantly Core 1–3 structures (Bergstrom et al., 2017).

Mucin *O*-glycans can be either branched or linear in structure, depending on their Core (e.g., Core 1 glycans are linear, and Core 2 glycans are branched; Podolsky, 1985; Li and Chai, 2019). *O*-glycans have been found to contain up to 20 residues and may include blood group determinants of ABO, Lewis groups, and glycan epitopes such as the linear antigen *i* (Gal*β*1-4GlcNAcβ1-3Gal; Vliegenthart, 2017). Fuc and NeuAc are monosaccharides decorating *O*-glycans in terminal *α*-linkages, and usually mucins contain sulfate groups. The latter two confer a negative charge on the mucin structure, crucial for selective mucin permeability and its rheological properties (De Weirdt and Van de Wiele, 2015). An acidic gradient, based on increasing amounts of NeuAc in mucins, has been shown in the gastrointestinal tract (Robbe et al., 2003).

Several studies have shown how alterations in mucin *O*-glycosylation patterns participate in disease. In cystic fibrosis patients, the respiratory epithelium shows higher degradation rates for MUC5B and MUC5AC, in addition to reduced sulfation, higher sialylation, and lower fucosylation (Schulz et al., 2007). This disease is characterized by chronic pulmonary infection and severe inflammation, and the changes mentioned above could be used as biomarkers. In the stomach, a broad diversity of *O*-glycans has been observed, but specific epitopes, such as Lewis b and α1-4GlcNAc, were found to promote adhesion of *Helicobacter pylori* to the mucus layer (Rossez et al., 2012; Jin et al., 2017). This pathogen is a direct cause of gastrointestinal ulcers and gastric cancer.

Similarly to mucins, glycoproteins in cancer cells show alterations in their *O*-glycosylation profiles. Truncated *O*-glycans such as a GalNAc residue with no further glycosylation (GalNAcα-Ser/Thr; Tn antigen) is considered a tumor marker given its presence is abnormal in glycoproteins (Itzkowitz et al., 1989). Sialylated Tn is also a common feature of cancer cells. An increase in core fucosylation is also observed in these cells. Consequently, these changes interfere with cell-cell adhesion processes, promoting tumor cell invasion (Pinho and Reis, 2015).

Importantly, *O*-linked glycans from human gastric mucin are structurally similar to those from the porcine stomach mucin. The oligosaccharides in both glycoproteins are mainly extended with Core 1 and Core 2 structures. The availability of porcine stomach mucin has facilitated the study of mucin function and its interactions with gut microorganisms. However, oligosaccharides in pig mucins have unique characteristics, like that they could contain extended Core 3 and Core 4 glycans (Padra et al., 2018). Pig gastric mucins are highly sulfated, usually terminating with galactose residues and have lower sialylation (Quintana-Hayashi et al., 2018).

Mucin glycosylation is in part mediated by gut microbiota. Using germ-free (GF) mice as control, it has been shown that the presence of certain members of gut microbiota is critical for the expression of glycosyltransferases participating in mucin *O*-glycosylation for example, ppGalNAcT, Core 1 *β*1,3-Galactosyltransferase (C1GALT1), and Fucosyltransferase (FUT2; Johansson et al., 2015). MUC2 *O*-linked glycans from colonic tissues of conventionally raised animals were more sialylated, fucosylated, and longer than GF mice (Arike et al., 2017). MUC2 from GF mice showed a reduced abundance of β1,6-*N*-acetyl glucosaminyltransferases, enzymes responsible for the formation of Core 2 and Core 4 *O*-glycans. These GF animals tended to produce shorter *O*-glycans (Arike et al., 2017). This result suggests that the gut microbiota modulates the *O*-glycosylation patterns of mucins, which in turn influence the composition the gut microbiota.

Mucin utilization by the gut microbiota is important for host health. It is known that animals that are fed mucin show higher fecal butyrate levels. This compound is an anti-inflammatory SCFA considered positive for health and significantly decreased in inflammatory diseases such as Ulcerative Colitis (UC; Chen et al., 2018; Yamada et al., 2019). In UC subjects, mucin *O*-glycan abundance correlates negatively with butyrate production in feces, showing a reduced utilization of *O*-glycans by the gut microbiota (Yamada et al., 2019). This study presented important *in vivo* evidence linking the foraging of host-derived glycans, the action of the gut microbiota, and inflammatory bowel diseases (IBDs). In a different context, mucin *O*-glycan consumption by gut microbes could be considered unfavorable for the host. Animals deprived of fiber in their diets show the promotion of gut inflammation and colitis. This was explained partly by their gut microbiota turning into endogenous mucin resources as the last carbon source available. Accessing the mucin layer by mucin degraders permits the colonization by *Citrobacter rodentium*, a mucosal pathogen of mice sharing several pathogenic features with human gastrointestinal pathogens such as enteropathogenic *Escherichia coli* (Collins et al., 2014; Desai et al., 2016).

Mucins and their *O*-glycans are essential in the gut barrier function. Modifications of their expression, organization, or glycosylation are likely to prevent this effect, as observed in IBD. The altered balance between pro-inflammatory cytokines (TNF, IL-1b, IL-8, and IL-17), anti-inflammatory cytokines (IL-4 and IL-13), and immuno-regulatory cytokines described in IBD is likely to modify mucin expression and glycosylation (Guan and Zhang, 2017). Furthermore, altered glycosylation and sulfation of colonic mucins in IBD subjects could alter the protective role of the colonic mucus barrier (Groux-Degroote et al., 2020).

### *O*-Linked Glycans From GMP

Glycomacropeptide is a constituent of whey (20–25% of the protein moiety), derived from *κ*-casein after chymosin treatment during cheesemaking (Manso and López-Fandiño, 2004; Sunds et al., 2019). GMP is a hydrophilic, negatively charged, 64 amino acid glycopeptide. The molecular weight of GMP ranges from 6.7 to 8 kDa (Córdova-Dávalos et al., 2019). GMP is available in large quantities in dairy streams (Rojas and Torres, 2013).

**Figure 2:**
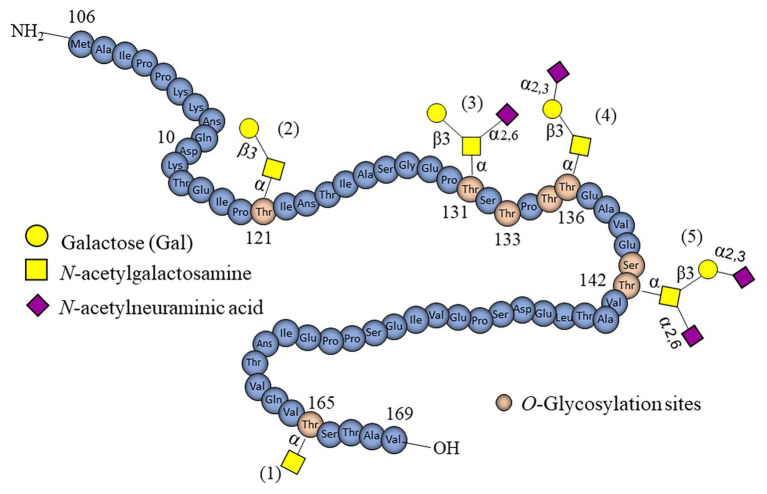
Schematic representation of *O*-linked glycans found in casein glycomacropeptide (GMP). Within the GMP structure, there are five identified *O*-glycans containing Gal, GalNAc, and NeuAc (named 1–5). Threonine residues serving as accepting glycosylation sites are also marked in brown.

The glycan portion is simpler compared to mucin oligosaccharides, containing only NeuAc, GalNAc, and Gal. GMP has five distinct *O*-linked glycans bound to Ser/Thr (Figure 2), corresponding to monosaccharides (0.8%), disaccharides (6.3%), trisaccharides (36.5%), and tetrasaccharides (56%; Saito and Itoh, 1992). The disaccharide GNB (Figure 2) is a building block found in GMP (Fiat and Jollès, 1989). Different *O*-glycosylation sites have been proposed in GMP, as determined by 2D gel electrophoresis and tandem mass spectrometry (Figure 2; Neelima et al., 2013). Thr in positions 121, 131, 133, 136, and 142 appear to be used as glycosylation sites, and positions 165, 135, and 141 have been proposed as potential sites (Thomä-Worringer et al., 2006). As expected, the functional and adhesion properties of GMP will depend on the position where *O*-glycans are attached, especially the sialylated. NeuAc is an acid sugar easily recognizable by mucin-degrading bacteria (Neelima et al., 2013).

## Utilization of Mucin *O*-Glycans by gut Microbes

While certain gut bacteria use mucin glycan as attachment sites, a few others go beyond and additionally deploy enzymatic activities to use mucin *O*-glycans as a nutrient (Tailford et al., 2015). Interestingly, this enzymatic capability is distributed in members across the four most dominant phyla in the gut microbiota (*Bacteroidetes*, *Actinobacteria*, *Firmicutes*, and *Verrucomicrobia*), suggesting it is an important trait for the gut microbiota.

Mucin *O*-glycan degradation by intestinal microorganisms requires an extensive array of glycosyl hydrolases (GHs), as expected from the complex structures, compositions, and diverse linkages found (Tailford et al., 2015). Most mucin-degrading bacteria base their strategies on exo-acting enzymes, including sialidases (GH33), fucosidases (GH29 and GH95), exo- and endo-*β*-*N*-acetylglucosaminidases (GH84 and GH85), β-galactosidases (GH2, GH20, and GH42), among others. Their activities imply sequential degradation. Other enzymes required for mucin utilization are *α*-*N*-acetylglucosaminidases (GH89) and α-*N*-acetylgalactosaminidases (GH101, GH129), which cleave a monosaccharide-peptide linkage after glycosidase activity. One potential exception to this exo-acting mechanism is a GH16 endoactive *O*-glycanase with mucin breakdown activities (Crouch et al., 2020). These activities release mono or disaccharides, which could be imported inside the bacterial cell or serve as cross-feeding metabolites (Turroni et al., 2018). Several enzymes related to mucin deglycosylation have been described, but the full mechanisms by which gut microbes utilize these glycans are unknown.

### Utilization of *O*-Linked Glycans by *Bacteroidetes* spp.

The *Bacteroides* genus is predominant in the adult gut microbiota, being generally considered as commensals. Genomic and functional studies in animals show that these species show a preference for utilizing complex carbohydrates (e.g., HMO), rather than simple carbohydrates (Wexler and Goodman, 2017). *Bacteroides* devote a large part of their genomes to polysaccharide utilization loci (PULs), which correlates with their broad diverse polysaccharide utilization. These gene clusters encode multiple extracellular GHs enzymes, oligosaccharide transporters, and binding proteins.

Early works studied the molecular system of complex glycan degradation in starch utilization in these species (Anderson and Salyers, 1989). *Bacteroides thetaiotaomicron* VPI-5482 is a model bacterium for mucin *O*-glycan utilization. The microorganism shows a remarkable growth in porcine mucin glycans (Martens et al., 2008). These oligosaccharides can induce the expression of at least 16 PULs in *Ba. thetaiotaomicron*, aiding in identifying loci involved in *O*-glycan utilization. These genes encode for putative glycolytic enzymes, including an α-L-fucosidase, endo-β-GlcNAc-ase, endo-*β*-galactosidase, α-GalNAc-ase, in addition to proteases, neuraminidase, and a sulfatase. The enzymes are suggested to play an orchestrated degradation of *O*-linked glycans (Sicard et al., 2017; Luis et al., 2018). The relevance of these genetic determinants has been confirmed *in vivo* experiments in GF mice, where mutants for these loci show reduced gut colonization.

**Figure 3:**
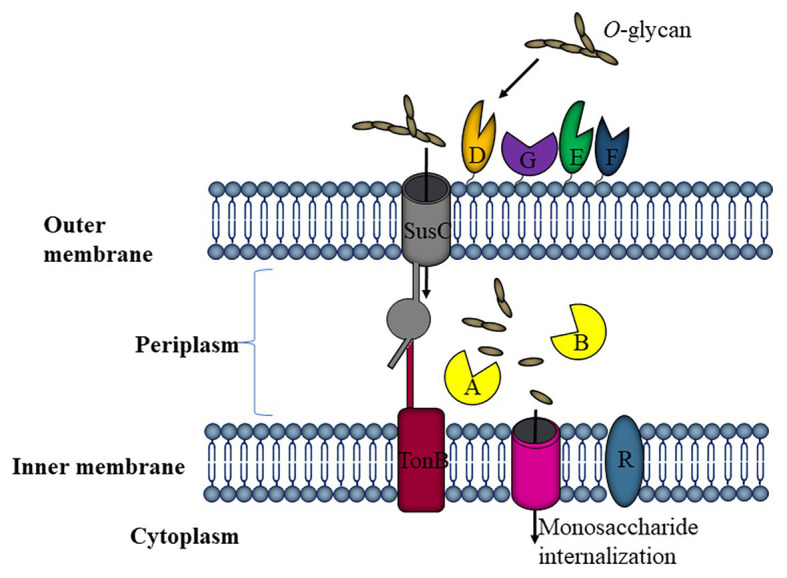
Representation of *O*-linked glycan utilization by *Bacteroides thetaiotaomicron*. The starch utilization system (Sus) is the machinery best described in *Ba. thetaiotaomicron* for the utilization of mucin-type *O*-glycans. SusDEF are recognition and binding sites for *O*-glycans, SusC is a TonB-dependent transporter for sugar importation, SusR is a predicted membrane-spanning regulator, and SusAB are glycosyl hydrolases (GHs; Zhang et al., 2018).

These enzymes are part of PULs also known as Starch utilization system (Sus), an operon of eight genes (SusRABCDEFG). Their products are predicted to locate in the periplasm or the outer membrane (Figure 3). Carbohydrates are metabolized in response to the SusR signal, which triggers the expression of hydrolyzing proteins. The processed oligosaccharides are transported by a TonB protein and are metabolized inside the cells (Cockburn and Koropatkin, 2016). SusD homologs possess a binding site for *O*-glycans (Tailford et al., 2015) and have been implicated in the utilization of *O*-linked mucin glycans. Interestingly, the transcriptional response mounted to utilize *O*-glycans is similar to that required for HMO consumption (Marcobal et al., 2011). Four PULs in *Ba. thetaiotaomicron* were found to be induced in HMO but not in the presence of mucin glycans, indicating that this microorganism can respond to building blocks in HMO not found in mucin. Regardless, the deletion of these PULs did not affect the ability of *Ba. thetaiotaomicron* to consume HMO. Apparently, for this strain *O*-glycans are not a premium substrate since several mono or simple carbohydrates repressed these genes (Pudlo et al., 2015). This was not observed for other *Bacteroides* species, indicating the complex regulation of glycan utilization and diversity on preference for carbon sources even at the species level (Pudlo et al., 2015).

Monocolonization of GF mice with *Ba. thetaiotaomicron* modulates cellular responses in mucin-producing goblet cells, increasing their differentiation and synthesis of mucins, changing their glycosylation patterns with a higher NeuAc content (Wrzosek et al., 2013). In the presence of *Faecalibacterium prausnitzii*, a keystone microbe and butyrate producer, these cellular responses were attenuated, probably maintaining epithelial homeostasis between cell lines.

*Bacteroides fragilis* is a commensal species, but certain strains could be enterotoxigenic causing serious diseases (Purcell et al., 2017; Chung et al., 2018). This species also has a PUL involved in the consumption of host *O*-glycans. This PUL is known as the commensal colonization factor (CCF; Lee et al., 2013). The genes within the CCF are homologous to *Ba. thetaiotaomicron* PULs, which are up-regulated possibly producing extracellular enzymes to sense and mediate *O*-glycan processing. These enzymes allow the commensal colonization of mucus, specifically in the crypts of the colon. Some of these have been characterized biochemically (Praharaj et al., 2018; Yamamoto et al., 2018).

### Utilization of *O*-Linked Glycans in *Firmicutes*

This phylum contains a broad diversity of genera, including commensal *Clostridium, Eubacterium*, and *Lactobacillus* species. In general, most *Firmicutes* in the gut microbiota prefer the assimilation of smaller rather than complex carbohydrates (Cockburn and Koropatkin, 2016; Ravcheev and Thiele, 2017). Several *Lactobacillus* species are endowed with mucin binding proteins (van Tassell and Miller, 2011), but they apparently lack any mucin utilization mechanism. However, within the *Firmicutes* a few members have acquired the ability to gain access to *O*-linked glycans, deploying several extracellular GHs.

A few pathogens in this group have evolved mechanisms for *O*-glycan utilization. *Clostridium perfringens*, an opportunistic pathogenic bacterium, can consume *N*-glycans and *O*-glycans from intestinal mucin by releasing extracellular glycosidases such as sialidases (Koutsioulis et al., 2008; Pluvinage et al., 2019). *Enterococcus faecalis* strains possess genes encoding endo-*β*-*N*-acetylglucosaminidase and endo-*α*-*N*-acetylgalactosaminidase (endo-α-GalNAc-ase) that release *N*-glycans. However, this pathobiont appears not to be able to utilize mucin *O*-linked glycans (Roberts et al., 2000; Morio et al., 2019). These microorganisms are not usually dominant in the gut microbiota, probably due to the barrier effect mounted by this community.

Commensal Firmicutes such as *Ruminococcus torques* and *Ruminococcus gnavus*, belonging to the *Lachnospiraceae* family, can also access the *O*-linked glycans from mucin, especially MUC2. *Ruminococcus torques* is endowed with an *α*-sialidase (GH33), α-fucosidase (GH29), *β*-galactosidase, β-*N*-acetylgalactosaminidase, β-*N*-acetylglucosaminidase, sialate *O*-acetylesterase, and glycosulfatase activities. Mucin deglycosylation is carried out from a mixture of exo and endo activities (Crost et al., 2016). *Ruminococcus gnavus* contains a similar enzymatic arsenal, but its strategy involves the chemical modification of NeuAc by a trans-sialidase, releasing 2,7-anhydro-Neu5Ac (Bell et al., 2019). Interestingly, this modification allows this microorganism to use this carbohydrate, making it inaccessible for others gut microbes (Bell et al., 2019). This strategy has proven essential for proper colonization of *R. gnavus* in mice.

*Ruminococcus gnavus* is part of the healthy gut microbiota but appears to be increased in IBDs (Hall et al., 2017). Similar to *Ba. thetaiotaomicron*, *R. gnavus* has the ability to modulate host cellular responses, especially genes encoding MUC2 and certain glycosyltransferases (Graziani et al., 2016). However, both *R. gnavus* and *R. torques* are increased several-fold in the intestinal mucosa of UC and Crohn’s disease subjects, suggesting that their mucolytic activities contribute to disease progression (Png et al., 2010).

Sheridan et al. (2016) used *in vitro* growth assays and comparative genomics to identify genes involved in general carbohydrate metabolism in 11 *Roseburia* spp. and *Eubacterium rectale* strains. These bacteria have been suggested next-generation probiotics (Lordan et al., 2019), considered desirable for the host due to the production of butyrate. Two *Roseburia inulinivorans* strains were found to contain a Gram-positive PUL (gpPUL) with putative mucin glycan degradation genes such as a desulfatase, four glycosidases, and an ATP-binding cassettes (ABC) transporter. While this evidence suggests certain butyrate-producers could use mucin *O*-glycans as a carbon source, none of the strains of this study was able to grow using type 2 or type 3 porcine-derived mucin. Further experiments are required to clarify the ability of these microorganisms to target mucin as carbon source and the role of the gpPUL.

### Utilization of *O*-Linked Glycans by *Bifidobacterium*

A few members of the phylum Actinobacteria can utilize glycans found in mucin, especially *Bifidobacterium* (Turroni et al., 2010; Katoh et al., 2017). This genus contains mostly commensal or beneficial microorganisms, dominant in the infant’s gut.

**Figure 4:**
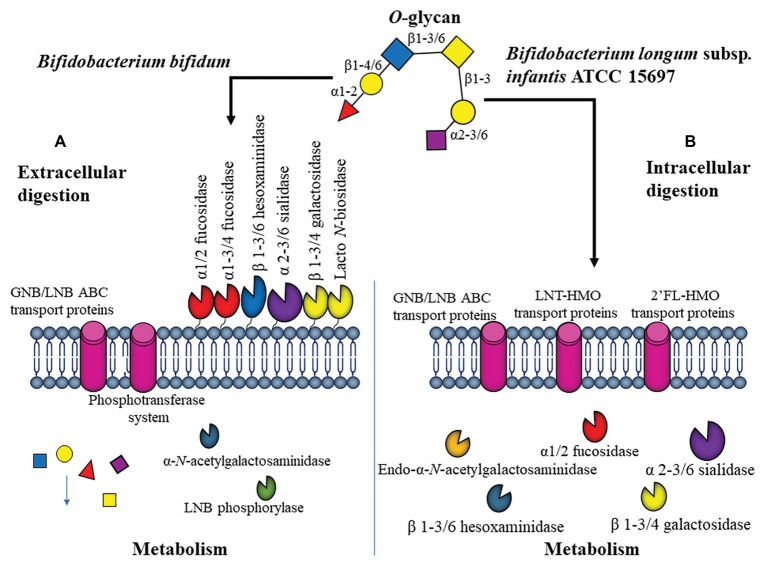
Representation of *O*-linked glycan utilization by *Bifidobacterium*: **(A)** *Bifidobacterium bifidum*, **(B)** *Bifidobacterium infantis*. Two major mechanisms of digestion of *O*-glycan utilization are presented, either extracellular to the left, or intracellular to the right (Garrido et al., 2012a).

Milani et al. (2016) showed that *Bi. bifidum* PRL2010 has a set of chromosomal loci that allows both *N*-glycans and *O*-glycan utilization. Seventy-seven genes, including GHs, glycosyltransferases, and glycosyl esterases were identified. *Bifidobacterium bifidum* contains a complete set of extracellular GHs, including *ex*o-*α*-sialidases, 1,2-α-L-fucosidase, 1,3/4-α-L-fucosidase, *N*-acetyl-β-hexosaminidases, and four β-galactosidases (Figure 4A). Several of these enzymes have been biochemically characterized, showing that they are membrane-bound and essential for mucin *O*-glycan assimilation (Shimada et al., 2015).

The consumption strategy of *Bi. bifidum* is mostly extracellular, where all these enzymes contain transmembrane domains and participate in mucin deglycosylation rendering GNB as a result. Apparently, *Bi. bifidum* does not utilize Fuc or NeuAc from complex glycans (Garrido et al., 2015). However, sialidase activity in this bacterium appears to promote bacterial adhesion to the epithelium (Nishiyama et al., 2017). This consumption mechanism allows the resulting mono and disaccharides to participate in cross-feeding with other microorganisms, for example, with *Bifidobacterium breve* and *Eubacterium hallii* (Egan et al., 2014; Bunesova et al., 2018).

The Lacto-N-Biose (LNB)/GNB cluster present in most bifidobacteria allows these microorganisms to consume Lacto-*N*-Tetraose from HMO and GNB resulting from *O*-glycan degradation. This cluster is present in several infant and adult-associated bifidobacteria. It contains an ABC transporter specific for these two disaccharides and intracellular enzymes for LNB/GNB utilization in the bifid shunt (Thompson et al., 2018).

A few enzymes in this genus have been characterized by their ability to target mucin glycans. Earlier works described an endo-α-GalNAc-ase in *Bi. longum* subsp. *longum* JCM 1217 (EngBF), which is a conserved extracellular GH101 enzyme in this subspecies, being highly specific for Core 1 *O*-glycans (Fujita et al., 2005). An α-acetylgalactosaminidase (NagBb) from *Bi. bifidum* JCM 1254 is a GH101 enzyme that hydrolyzes *O*-glycans from Core 1 and Core 3 from mucin MUC2 (Kiyohara et al., 2012). The enzyme shows a high affinity for the Tn antigen (GalNAcα-Ser/Thr). Finally, a GH89 from the same microorganism shows affinity for GlcNAcα1-4Gal, an epitope commonly found in terminal positions in mucin glycans (Shimada et al., 2015). All these enzymes have great potential as a tool in glycobiology studies, and the obtention of prebiotics from complex mucin sources. Finally, evidence regarding the utilization of mucin *O*-glycans by *Bifidobacterium* has primarily been studied *in vitro*, and further studies are required to understand the implications of these strategies for the host. *Bi. longum*, as well as inulin, show protective effects against mucus deterioration caused by a mucin-eroding gut microbiota (Schroeder et al., 2018).

### Utilization of *O*-Linked Glycans by *Verrucomicrobia*

In recent years, *A. muciniphila* has received considerable attention for its associations with health. Its abundance inversely correlates with the onset of IBDs and metabolic disorders (Dao et al., 2016; Lopez-Siles et al., 2018; Yassour et al., 2018), and it has been suggested to play an anti-inflammatory role (Cani and de Vos, 2017). It has also been proposed as a candidate next-generation probiotic, which in addition to the modulation of host responses possesses several technological properties compatible with scale-up processes (Cani and de Vos, 2017). Recently, the administration of pasteurized doses of *A. muciniphila* to overweight individuals improved certain inflammation and metabolic markers, being well-tolerated and safe (Depommier et al., 2019).

*Akkermansia muciniphila* is a Gram-negative bacterium that specializes in using mucins as nitrogen and carbon sources. This contrasts with other generalist gut microbes such as *Bacteroides* spp., which can target multiple sources for growth. Its specificity for mucin also presents a remarkable example of microbial adaptations to host conditions. *Akkermansia muciniphila* is the only member of the phylum *Verrucomicrobia* cultured and found in the gut microbiota, representing the 3% of intestinal bacteria detected in adult feces (Everard et al., 2014). Despite being considered a strict anaerobic microbe, recent studies have determined that it colonizes the mucus layer near epithelial cells, characterized by microaerophilic levels of oxygen (Ouwerkerk et al., 2016). *Akkermansia muciniphila* cannot synthesize Thr; however, it obtains this amino acid from mucin, which is one of the most abundant amino acids in its structure (van der Ark et al., 2018).

This microorganism is underrepresented in IBD, especially UC patients. Yamada et al. (2019) reported that fermentation of mucin *O*-glycans is associated with the production of butyrate in healthy subjects. However, subjects with UC show altered mucin *O*-glycosylation patterns, and the abundance of *A. muciniphila* was inversely proportional to the markers of inflammation (Earley et al., 2019; Yamada et al., 2019). This suggests that the *O*-glycosylation patterns present in mucins influence the anti-inflammatory activity of certain commensal microorganisms.

Studies show that the mucolytic activity and metabolite production by *A. muciniphila* promotes beneficial microbial networks (Belzer et al., 2017). Mucin degradation by gut microorganisms might be considered a pathogenic trait because it reduces the protective layer of the complex mucus layer. Intriguingly, in IL10-genetically deficient mice *A. muciniphila* contributes to colitis (Seregin et al., 2017). However, these degrading microorganisms may stimulate mucin accumulation, renewal, and thickening of the mucosal layer. In addition, the complexity of the mucin *O*-glycans makes these structures protective and inaccessible to most bacteria, except those that have developed consumption mechanisms such as *A. muciniphila* and *Ba. thetaiotaomicron*.

The genome analysis of *A. muciniphila* ATCC BAA-835, isolated from the human gastrointestinal tract, predicted the presence of a large number of mucinases (van Passel et al., 2011). This is a general term referring to GHs or lyases with combined activity against mucin. It was also reported that 26% of its proteome contains a signal peptide site, indicating a preference for extracellular degradation of macromolecules. Consequently, the putative strategy employed by this microorganism is to secrete an extensive array of extracellular proteins that hydrolyze *N*-glycans and *O*-glycans in simple sugars, some of which are later internalized (Shin et al., 2019). Regarding its enzymatic machinery, *A. muciniphila* is predicted to contain *α*/*β*-D-galactosidase, α-L-fucosidase, α/β-*N*-acetylgalactosaminidase, two α-*N*-Acetyl-glucosaminidases, neuraminidase, and a sulfatase. Few studies have confirmed the activities of some of these enzymes (Wang et al., 2018; Shin et al., 2019; Meng et al., 2020), including sulfatases and proteases targeting MUC2. Major end-metabolites produced by this bacterium are propionate, acetate, and sulfate (Ottman et al., 2017).

## Utilization of Gmp-Derived Glycans by gut Microbes

Glycomacropeptide is a highly sialylated glycopeptide resulting from the cheesemaking process. It is an abundant source of *O*-glycans. However, not much attention has been paid to its potential applications for promoting a healthy gut microbiota. Compared to mucin, most studies have focused on the utilization of GMP by probiotic microorganisms *in vitro*, sometimes with contradictory results (Córdova-Dávalos et al., 2019). Early work from Idota et al. (1994) showed that certain *Bifidobacterium*, including *Bi. bifidum*, *Bi. breve*, *Bi. longum*, and *Bifidobacterium adolescentis* could grow on GMP with OD_600nm_ values ranging from 0.7 to 2.70. Although purified GMP contains small amounts of lactose, which could contribute to the above results, the latter two microorganisms do not have the sialidases required to hydrolyze the GMP *O*-glycans (O’Callaghan and van Sinderen, 2016).

*Bifidobacterium longum* subsp. *infantis* ATCC 15697 (*Bifidobacterium infantis*) is a dominant bacterium in the gut microbiota of newborns. It has been well characterized by its ability to fully utilize several HMO as a carbon source, with protective effects on the infant (Thompson et al., 2018; Henrick et al., 2019). The utilization strategy for this microorganism relies on ABC for internalization of intact glycans, subsequently degraded by intracellular GHs (Figure 4B; Zúñiga et al., 2018). *Bifidobacterium infantis* shows a preference for host glycans containing within its genome 16 GH, including several *α*-fucosidases, β-galactosidases, β-hexosaminidases, and α-sialidases (Thompson et al., 2018). This microorganism has also evolved determinants and mechanisms for the vigorous utilization of milk-derived *N*-glycans (Garrido et al., 2012b; Karav et al., 2016).

Considering the structural similarity between HMO, *N*-glycans, and *O*-glycans, it could be expected that this microorganism also targets mucins as a growth substrate. Interestingly, *B. infantis* is not able to access the *O*-glycans of these glycoproteins (Kim et al., 2013; Turroni et al., 2018). Most glycosidases in *B. infantis* are intracellular, and it lacks homologs to α-*N*-Acetylgalactosaminidases found in *B. bifidum* and *B. longum*.

Strikingly, *B. infantis* has been shown to grow vigorously using GMP (O’Riordan et al., 2018). A 20.6 ± 3.6% increase in OD_600nm_ was observed in the mid-exponential phase of *B. longum* ssp. *infantis* cultures supplemented with GMP, indicating a growth-promoting effect. *In vitro*, it has been shown that *B. infantis* growth is proportional to GMP concentration in the culture media. GMP periodate treatment (GMP-P) inactivates the glycan portion of the peptide chain, and growth was compared with native GMP. GMP-P resulted in a substantially lower (5.5%) increase in growth compared with full GMP in the mid-exponential phase. Under these conditions, *B. infantis* was not able to grow using GMP-P, indicating that the GMP *O*-glycan portion is essential in *B. infantis* growth.

Transcriptomic analysis of the GMP utilization revealed the induction of two intracellular GH25 enzymes, a family related to bacterial lysozymes, and a solute binding protein from an ABC transporter (O’Riordan et al., 2018). These results provide new insights regarding the adaptations of this probiotic microorganism for a milk-derived substrate. However, how this infant-gut associated bacterium can use GMP *O*-glycans as the sole carbon source is not clear, especially considering it cannot access mucin *O*-glycans. It is possible that GMP, a smaller and structurally and physicochemical simpler molecule, is easier to access. Another possibility is the participation of proteases, which could facilitate the import of GMP-derived *O*-glycans into the bacterial cell for intracellular processing.

## Conclusion

*O*-linked glycans are complex oligosaccharides decorating host glycoproteins. They are produced endogenously in mucins or could be found in secretions such as milk. These glycans are accessed, released, and consumed by individual members of the gut microbiota, especially beneficial gut microbes such as *Bifidobacterium*, *Bacteroides*, *Akkermansia*, and stimulate the growth of next-generation probiotics such as *Roseburia* and other butyrate-producing bacteria.

*O*-glycans serve as signaling molecules for cell secretion and provide greater resistance to proteolysis (Boutrou et al., 2008). Under conditions of dietary fiber depletion, endogenous mucin degradation occurs associated with microbial activity, promoting inflammation, and intestinal disease.

*O*-glycans derived from milk glycopeptides such as in GMP are an attractive opportunity to use them in foods as emerging prebiotics, promoting a healthy gut microbiota. Unfortunately, there are still several limitations to this goal, and no human studies have evaluated its prebiotic effect. While, we have advanced in understanding some of the molecular mechanisms involved in *O*-linked glycan utilization in single microorganisms, the impact of this utilization in metabolic interactions and networks has been evaluated only in a small number of studies.

These gaps have been hindered in part because we lack enzymes or other tools to recover full *O*-glycans from dairy streams or mucin sources. In contrast, certain enzymes have been described for the recovery of *N*-glycans from dairy byproducts. This offers an opportunity for identifying novel enzymes from gut microbes or improving the activity of current endo-α-*N*-acetylgalactosaminidases.

Moreover, the consequences for the host of the microbial utilization of *O*-glycans, or glycoproteins containing *O*-glycans, have not been evaluated and remains a critical question. Whether gut microbes accessing the mucus layer and releasing *O*-glycans is beneficial for the host is still unclear. Furthermore, the study of *O*-glycans utilization by the gut microbiota, and how these microbes shape mucin glycosylation patterns, should be further studied in the context of intestinal inflammation and IBDs, as well as cancer progression.

## Author Contributions

KG-M, MV-S, and DG conceived and wrote the manuscript. KG-M prepared the Figures. DG critically reviewed the manuscript. All authors approved the final version of the manuscript.

### Conflict of Interest

The authors declare that the research was conducted in the absence of any commercial or financial relationships that could be construed as a potential conflict of interest.

## References

[ref1] Anderson K. L. Salyers A. A. (1989). Biochemical evidence that starch breakdown by *Bacteroides thetaiotaomicron* involves outer membrane starch-binding sites and periplasmic starch-degrading enzymes. J. Bacteriol. 171, 3192–3198. 10.1128/JB.171.6.3192-3198.1989, PMID: 2722747 PMC210036

[ref2] Arike L. Hansson G. C. (2016). The densely *O*-glycosylated MUC2 mucin protects the intestine and provides food for the commensal bacteria. J. Mol. Biol. 428, 3221–3229. 10.1016/j.jmb.2016.02.010, PMID: 26880333 PMC4982847

[ref3] Arike L. Holmén-Larsson J. Hansson G. C. (2017). Intestinal Muc2 mucin *O*-glycosylation is affected by microbiota and regulated by differential expression of glycosyltranferases. Glycobiology 27, 318–328. 10.1093/glycob/cww134, PMID: 28122822 PMC5444243

[ref4] Atuma C. Strugala V. Allen A. Holm L. (2001). The adherent gastrointestinal mucus gel layer: thickness and physical state in vivo. Am. J. Physiol. Gastrointest. Liver Physiol. 280, G922–G929. 10.1152/ajpgi.2001.280.5.G922, PMID: 11292601

[ref5] Bansil R. Turner B. S. (2018). The biology of mucus: composition, synthesis and organization. Adv. Drug Deliv. Rev. 124, 3–15. 10.1016/j.addr.2017.09.023, PMID: 28970050

[ref6] Baum L. G. Cobb B. A. (2016). The direct and indirect effects of glycans on immune function. Glycobiology 27, 619–624. 10.1093/glycob/cwx036, PMID: 28460052

[ref7] Bell A. Brunt J. Crost E. Vaux L. Nepravishta R. Owen C. D.. (2019). Elucidation of a sialic acid metabolism pathway in mucus-foraging *Ruminococcus gnavus* unravels mechanisms of bacterial adaptation to the gut. Nat. Microbiol. 4, 2393–2404. 10.1038/s41564-019-0590-7, PMID: 31636419 PMC6881182

[ref8] Belzer C. Chia L. W. Aalvink S. Chamlagain B. Piironen V. Knol J.. (2017). Microbial metabolic networks at the mucus layer lead to diet-independent butyrate and vitamin B12 production by intestinal symbionts. mBio 8, e00770–e00717. 10.1128/mBio.00770-17, PMID: 28928206 PMC5605934

[ref350] BeMiller J. N. (2019). “Oligosaccharides” in Carbohydrate chemistry for food scientists. ed. BeMiller J. N. (PA: Woodhead Publishing and AACC International Press (Elsevier)), 49–74.

[ref9] Brockhausen I. Stanley P. (2017). “O-GalNAc glycans” in Essentials of glycobiology. eds. Varki A. Cummings R. D. Esko J. D. Stanley P. Hart G. W. Aebi M. (NY: Cold Spring Harbor Laboratory Press). 20301232

[ref10] Bergstrom K. Fu J. Johansson M. E. V. Liu X. Gao N. Wu Q.. (2017). Core 1– and 3–derived *O*-glycans collectively maintain the colonic mucus barrier and protect against spontaneous colitis in mice. Mucosal Immunol. 10, 91–103. 10.1038/mi.2016.45, PMID: 27143302 PMC5097036

[ref11] Bergstrom K. Xia L. (2013). Mucin-type *O*-glycans and their roles in intestinal homeostasis. Glycobiology 23, 1026–1037. 10.1093/glycob/cwt045, PMID: 23752712 PMC3858029

[ref12] Bernard L. Bonnet M. Delavaud C. Delosière M. Ferlay A. Fougère H. (2018). Milk fat globule in ruminant: major and minor compounds, nutritional regulation and differences among species. Eur. J. Lipid Sci. Technol. 120:1700039. 10.1002/ejlt.201700039

[ref13] Boutrou R. Jardin J. Blais A. Tomé D. Léonil J. (2008). Glycosylations of κ-casein-derived caseinomacropeptide reduce its accessibility to endo- but not exointestinal brush border membrane peptidases. J. Agric. Food Chem. 56, 8166–8173. 10.1021/jf801140d, PMID: 18698795

[ref14] Brockhausen I. Stanley P. (2017). “O-GalNAc glycans” in Essentials of glycobiology. eds. Varki A. Cummings R. D. Esko J. D. Stanley P. Hart G. W. Aebi M. (NY: Cold Spring Harbor Laboratory Press). 20301232

[ref15] Brosseau C. Selle A. Palmer D. J. Prescott S. L. Barbarot S. Bodinier M. (2019). Prebiotics: mechanisms and preventive effects in allergy. Nutrients 11:1841. 10.3390/nu11081841, PMID: 31398959 PMC6722770

[ref16] Bunesova V. Lacroix C. Schwab C. (2018). Mucin cross-feeding of infant bifidobacteria and *Eubacterium hallii*. Microb. Ecol. 75, 228–238. 10.1007/s00248-017-1037-4, PMID: 28721502

[ref17] Cani P. D. de Vos W. M. (2017). Next-generation beneficial microbes: the case of *Akkermansia muciniphila*. Front. Microbiol. 8:1765. 10.3389/fmicb.2017.01765, PMID: 29018410 PMC5614963

[ref18] Chen G. Ran X. Li B. Li Y. He D. Huang B.. (2018). Sodium butyrate inhibits inflammation and maintains epithelium barrier integrity in a TNBS-induced inflammatory bowel disease mice model. EBioMedicine 30, 317–325. 10.1016/j.ebiom.2018.03.030, PMID: 29627390 PMC5952406

[ref19] Chung L. Thiele Orberg E. Geis A. L. Chan J. L. Fu K. DeStefano Shields C. E.. (2018). *Bacteroides fragilis* toxin coordinates a pro-carcinogenic inflammatory cascade via targeting of colonic epithelial cells. Cell Host Microbe 23, 203.e5–214.e5. 10.1016/j.chom.2018.01.007, PMID: 29398651 PMC5954996

[ref20] Cockburn D. W. Koropatkin N. M. (2016). Polysaccharide degradation by the intestinal microbiota and its influence on human health and disease. J. Mol. Biol. 428, 3230–3252. 10.1016/j.jmb.2016.06.021, PMID: 27393306

[ref21] Collins J. W. Keeney K. M. Crepin V. F. Rathinam V. A. K. Fitzgerald K. A. Finlay B. B.. (2014). *Citrobacter rodentium*: infection, inflammation and the microbiota. Nat. Rev. Microbiol. 12, 612–623. 10.1038/nrmicro3315, PMID: 25088150

[ref22] Córdova-Dávalos L. Jiménez M. Salinas E. (2019). Glycomacropeptide bioactivity and health: a review highlighting action mechanisms and signaling pathways. Nutrients 11:598. 10.3390/nu11030598, PMID: 30870995 PMC6471465

[ref23] Corfield A. P. (2000). Mucins and mucosal protection in the gastrointestinal tract: new prospects for mucins in the pathology of gastrointestinal disease. Gut 47, 589–594. 10.1136/gut.47.4.589, PMID: 10986224 PMC1728059

[ref24] Corfield A. P. (2015). Mucins: a biologically relevant glycan barrier in mucosal protection. Biochim. Biophys. Acta 1850, 236–252. 10.1016/j.bbagen.2014.05.003, PMID: 24821013

[ref25] Crost E. H. Tailford L. E. Monestier M. Swarbreck D. Henrissat B. Crossman L. C.. (2016). The mucin-degradation strategy of *Ruminococcus gnavus*: the importance of intramolecular trans-sialidases. Gut Microbes 7, 302–312. 10.1080/19490976.2016.1186334, PMID: 27223845 PMC4988440

[ref26] Crouch L. I. Liberato M. V. Urbanowicz P. A. Baslé A. Lamb C. A. Stewart C. J.. (2020). Prominent members of the human gut microbiota express endo-acting *O*-glycanases to initiate mucin breakdown. Nat. Commun. 11:4017. 10.1038/s41467-020-17847-5, PMID: 32782292 PMC7419316

[ref27] Dao M. C. Everard A. Aron-Wisnewsky J. Sokolovska N. Prifti E. Verger E. O.. (2016). *Akkermansia muciniphila* and improved metabolic health during a dietary intervention in obesity: relationship with gut microbiome richness and ecology. Gut 65, 426–436. 10.1136/gutjnl-2014-308778, PMID: 26100928

[ref28] Davis J. C. C. Totten S. M. Huang J. O. Nagshbandi S. Kirmiz N. Garrido D. A.. (2016). Identification of oligosaccharides in feces of breast-fed infants and their correlation with the gut microbial community. Mol. Cell. Proteomics 15, 2987–3002. 10.1074/mcp.M116.060665, PMID: 27435585 PMC5013312

[ref30] Demouveaux B. Gouyer V. Gottrand F. Narita T. Desseyn J. L. (2018). Gel-forming mucin interactome drives mucus viscoelasticity. Adv. Colloid Interface Sci. 252, 69–82. 10.1016/j.cis.2017.12.005, PMID: 29329667

[ref31] Depommier C. Everard A. Druart C. Plovier H. Van Hul M. Vieira-Silva S.. (2019). Supplementation with *Akkermansia muciniphila* in overweight and obese human volunteers: a proof-of-concept exploratory study. Nat. Med. 25, 1096–1103. 10.1038/s41591-019-0495-2, PMID: 31263284 PMC6699990

[ref32] Desai M. S. Seekatz A. M. Koropatkin N. M. Kamada N. Hickey C. A. Wolter M.. (2016). A dietary fiber-deprived gut microbiota degrades the colonic mucus barrier and enhances pathogen susceptibility. Cell 167, 1339.e21–1353.e21. 10.1016/j.cell.2016.10.043, PMID: 27863247 PMC5131798

[ref29] De Weirdt R. Van de Wiele T. (2015). Micromanagement in the gut: microenvironmental factors govern colon mucosal biofilm structure and functionality. NPJ Biofilms Microbiomes 1:15026. 10.1038/npjbiofilms.2015.26, PMID: 28721237 PMC5515210

[ref33] Dhanisha S. S. Guruvayoorappan C. Drishya S. Abeesh P. (2018). Mucins: structural diversity, biosynthesis, its role in pathogenesis and as possible therapeutic targets. Crit. Rev. Oncol. Hematol. 122, 98–122. 10.1016/j.critrevonc.2017.12.006, PMID: 29458795

[ref34] Donovan S. M. (2019). “Human milk proteins: composition and physiological significance” in Human milk: composition, clinical benefits and future opportunities. eds. Donovan S. M. German J. B. Lönnerdal B. Lucas A. (Switzerland: Nestlé Nutrition Institute/Basel: S. Karger AG), 93–101. 10.1159/000490298 30865978

[ref35] Earley H. Lennon G. Balfe Á. Coffey J. C. Winter D. C. O’Connell P. R. (2019). The abundance of *Akkermansia muciniphila* and its relationship with sulphated colonic mucins in health and ulcerative colitis. Sci. Rep. 9:15683. 10.1038/s41598-019-51878-3, PMID: 31666581 PMC6821857

[ref36] Egan M. O’Connell Motherway M. Kilcoyne M. Kane M. Joshi L. Ventura M.. (2014). Cross-feeding by *Bifidobacterium breve* UCC2003 during co-cultivation with *Bifidobacterium bifidum* PRL2010 in a mucin-based medium. BMC Microbiol. 14:282. 10.1186/s12866-014-0282-7, PMID: 25420416 PMC4252021

[ref37] Everard A. Lazarevic V. Gaïa N. Johansson M. Ståhlman M. Backhed F.. (2014). Microbiome of prebiotic-treated mice reveals novel targets involved in host response during obesity. ISME J. 8, 2116–2130. 10.1038/ismej.2014.45, PMID: 24694712 PMC4163056

[ref38] Fiat A. M. Jollès P. (1989). Caseins of various origins and biologically active casein peptides and oligosaccharides: structural and physiological aspects. Mol. Cell. Biochem. 87, 5–30. 10.1007/BF00421079, PMID: 2671666

[ref39] Fujita K. Fusako O. Nagamine N. Katayama T. Hiratake J. Sakata K.. (2005). Identification and molecular cloning of a novel glycoside hydrolase family of core 1 type *O*-glycan-specific endo-α-*N*-acetylgalactosaminidase from *Bifidobacterium longum*. J. Biol. Chem. 280, 37415–37422. 10.1074/jbc.M506874200, PMID: 16141207

[ref40] Garrido D. Barile D. Mills D. A. (2012a). A molecular basis for bifidobacterial enrichment in the infant gastrointestinal tract. Adv. Nutr. 3, 415S–421S. 10.3945/an.111.001586, PMID: 22585920 PMC3649478

[ref41] Garrido D. Nwosu C. Ruiz-Moyano S. Aldredge D. German J. B. Lebrilla C. B.. (2012b). Endo-β-*N*-acetylglucosaminidases from infant gut-associated bifidobacteria release complex *N*-glycans from human milk glycoproteins. Mol. Cell. Proteomics 11, 775–785. 10.1074/mcp.M112.018119, PMID: 22745059 PMC3434770

[ref42] Garrido D. Ruiz-Moyano S. Lemay D. G. Sela D. A. German J. B. Mills D. A. (2015). Comparative transcriptomics reveals key differences in the response to milk oligosaccharides of infant gut-associated bifidobacteria. Sci. Rep. 5:13517. 10.1038/srep13517, PMID: 26337101 PMC4559671

[ref43] Gibson G. R. McCartney A. L. Rastall R. A. (2005). Prebiotics and resistance to gastrointestinal infections. Br. J. Nutr. 93, S31–S34. 10.1079/BJN20041343, PMID: 15877892

[ref44] Gibson G. R. Probert H. M. Loo J. V. Rastall R. A. Roberfroid M. B. (2017). Dietary modulation of the human colonic microbiota: updating the concept of prebiotics. Nutr. Res. Rev. 14, 259–275. 10.1079/NRR200479, PMID: 19079930

[ref45] Graziani F. Pujol A. Nicoletti C. Dou S. Maresca M. Giardina T.. (2016). *Ruminococcus gnavus* E1 modulates mucin expression and intestinal glycosylation. J. Appl. Microbiol. 120, 1403–1417. 10.1111/jam.13095, PMID: 26868655

[ref46] Groux-Degroote S. Cavdarli S. Uchimura K. Allain F. Delannoy P. (2020). Glycosylation changes in inflammatory diseases. Adv. Protein Chem. Struct. Biol. 119, 111–156. 10.1016/bs.apcsb.2019.08.008, PMID: 31997767

[ref47] Guan Q. Zhang J. (2017). Recent advances: the imbalance of cytokines in the pathogenesis of inflammatory bowel disease. Mediators Inflamm. 2017:4810258. 10.1155/2017/4810258, PMID: 28420941 PMC5379128

[ref48] Hall A. B. Yassour M. Sauk J. Garner A. Jiang X. Arthur T.. (2017). A novel *Ruminococcus gnavus* clade enriched in inflammatory bowel disease patients. Genome Med. 9:103. 10.1186/s13073-017-0490-5, PMID: 29183332 PMC5704459

[ref49] Henrick B. M. Chew S. Casaburi G. Brown H. K. Frese S. A. Zhou Y.. (2019). Colonization by *B. infantis* EVC001 modulates enteric inflammation in exclusively breastfed infants. Pediatr. Res. 86, 749–757. 10.1038/s41390-019-0533-2, PMID: 31443102 PMC6887859

[ref50] Idota T. Kawakami H. Nakajima I. (1994). Growth-promoting effects of *N*-acetylneuraminic acid-containing substances on bifidobacteria. Biosci. Biotechnol. Biochem. 58, 1720–1722. 10.1271/bbb.58.1720

[ref51] Itano N. (2019). Implications of altered *O*-glycosylation in tumour immune evasion. J. Biochem. 165, 387–390. 10.1093/jb/mvz003, PMID: 30649348

[ref52] Itzkowitz S. H. Yuan M. Montgomery C. K. Kjeldsen T. Takahashi H. K. Bigbee W. L.. (1989). Expression of Tn, sialosyl-Tn, and T antigens in human colon cancer. Cancer Res. 49, 197–204. PMID: 2908846

[ref53] JinC.KennyD. T.SkoogE. C.PadraM.AdamczykB.VitizevaV.. (2017). Structural diversity of human gastric mucin glycans. Mol. Cell. Proteomics 16, 743–758. 10.1074/mcp.M117.067983, PMID: 28461410

[ref54] Johansson M. E. V. Jakobsson H. E. Holmén-Larsson J. Schütte A. Ermund A. Rodríguez-Piñeiro A. M.. (2015). Normalization of host intestinal mucus layers requires long-term microbial colonization. Cell Host Microbe 18, 582–592. 10.1016/j.chom.2015.10.007, PMID: 26526499 PMC4648652

[ref55] Karav S. Le Parc A. Nobrega de Moura Bell J. M. L. Frese S. A. Kirmiz N. Block D. E.. (2016). Oligosaccharides released from milk glycoproteins are selective growth substrates for infant-associated bifidobacteria. Appl. Environ. Microbiol. 82, 3622–3630. 10.1128/AEM.00547-16, PMID: 27084007 PMC4959171

[ref56] Katoh T. Maeshibu T. Kikkawa K. Gotoh A. Tomabechi Y. Nakamura M.. (2017). Identification and characterization of a sulfoglycosidase from *Bifidobacterium bifidum* implicated in mucin glycan utilization. Biosci. Biotechnol. Biochem. 81, 2018–2027. 10.1080/09168451.2017.1361810, PMID: 28814130

[ref57] Kesimer M. Sheehan J. K. (2012). Mass spectrometric analysis of mucin core proteins. Methods Mol. Biol. 842, 67–79. 10.1007/978-1-61779-513-8_4, PMID: 22259130 PMC5151179

[ref58] Kim J. -H. An H. J. Garrido D. German J. B. Lebrilla C. B. Mills D. A. (2013). Proteomic analysis of *Bifidobacterium longum* subsp. infantis reveals the metabolic insight on consumption of prebiotics and host glycans. PLoS One 8:e57535. 10.1371/journal.pone.0057535, PMID: 23469017 PMC3582569

[ref59] Kirmiz N. Robinson R. C. Shah I. M. Barile D. Mills D. A. (2018). Milk glycans and their interaction with the infant-gut microbiota. Annu. Rev. Food Sci. Technol. 9, 429–450. 10.1146/annurev-food-030216-030207, PMID: 29580136 PMC5999319

[ref60] Kiyohara M. Nakatomi T. Kurihara S. Fushinobu S. Suzuki H. Tanaka T.. (2012). α-*N*-acetylgalactosaminidase from infant-associated bifidobacteria belonging to novel glycoside hydrolase family 129 is implicated in alternative mucin degradation pathway. J. Biol. Chem. 287, 693–700. 10.1074/jbc.M111.277384, PMID: 22090027 PMC3249124

[ref61] Koutsioulis D. Landry D. Guthrie E. P. (2008). Novel endo-α-*N*-acetylgalactosaminidases with broader substrate specificity. Glycobiology 18, 799–805. 10.1093/glycob/cwn069, PMID: 18635885 PMC2553423

[ref62] Lamacchia C. Musaico D. Henderson M. E. Bergillos-meca T. Roul M. Landriscina L. (2018). Temperature-treated gluten proteins in gluten-friendly™ bread increase mucus production and gut-barrier function in human intestinal goblet cells. J. Funct. Foods 48, 507–514. 10.1016/j.jff.2018.07.047

[ref64] Lee S. M. Donaldson G. P. Mikulski Z. Boyajian S. Ley K. Mazmanian S. K. (2013). Bacterial colonization factors control specificity and stability of the gut microbiota. Nature 501, 426–429. 10.1038/nature12447, PMID: 23955152 PMC3893107

[ref63] Le Parc A. Karav S. Maria J. Nobrega L. Bell D. M. Frese S. A.. (2015). A novel endo-β-*N*-acetylglucosaminidase releases specific *N*-glycans depending on different reaction conditions. Biotechnol. Prog. 31, 1323–1330. 10.1002/btpr.2133, PMID: 26101185 PMC4623945

[ref65] Li Z. Chai W. (2019). Mucin *O*-glycan microarrays. Curr. Opin. Struct. Biol. 56, 187–197. 10.1016/j.sbi.2019.03.032, PMID: 31063936

[ref66] Lillehoj E. P. Kato K. Lu W. Kim K. C. (2013). Cellular and molecular biology of airway mucins. Int. Rev. Cell Mol. Biol. 303, 139–202. 10.1016/B978-0-12-407697-6.00004-0, PMID: 23445810 PMC5593132

[ref67] Lopez-Siles M. Enrich-Capó N. Aldeguer X. Sabat-Mir M. Duncan S. H. Garcia-Gil L. J.. (2018). Alterations in the abundance and co-occurrence of *Akkermansia muciniphila* and *Faecalibacterium prausnitzii* in the colonic mucosa of inflammatory bowel disease subjects. Front. Cell. Infect. Microbiol. 8:281. 10.3389/fcimb.2018.00281, PMID: 30245977 PMC6137959

[ref68] Lordan C. Thapa D. Ross R. P. Cotter P. D. (2019). Potential for enriching next-generation health-promoting gut bacteria through prebiotics and other dietary components. Gut Microbes 11, 1–20. 10.1080/19490976.2019.1613124, PMID: 31116628 PMC6973326

[ref69] Luis A. S. Briggs J. Zhang X. Farnell B. Ndeh D. Labourel A.. (2018). Dietary pectic glycans are degraded by coordinated enzyme pathways in human colonic *Bacteroides*. Nat. Microbiol. 3, 210–219. 10.1038/s41564-017-0079-1, PMID: 29255254 PMC5784806

[ref70] Magalhães A. Rossez Y. Robbe-Masselot C. Maes E. Gomes J. Shevtsova A.. (2016). MUC5AC gastric mucin glycosylation is shaped by FUT2 activity and functionally impacts *Helicobacter pylori* binding. Sci. Rep. 6:25575. 10.1038/srep25575, PMID: 27161092 PMC4861914

[ref71] Magnelli P. E. Bielik A. M. Guthrie E. P. (2011). Identification and characterization of protein glycosylation using specific endo- and exoglycosidases. J. Vis. Exp. 58:e3749. 10.3791/3749, PMID: 22230788 PMC3369641

[ref72] Manso M. A. López-Fandiño R. (2004). κ-casein macropeptides from cheese whey: physicochemical, biological, nutritional, and technological features for possible uses. Food Rev. Int. 20, 329–355. 10.1081/FRI-200033456

[ref73] Marcobal A. Barboza M. Sonnenburg E. D. Pudlo N. Martens E. C. Desai P.. (2011). Bacteroides in the infant gut consume milk oligosaccharides via mucus-utilization pathways. Cell Host Microbe 10, 507–514. 10.1016/j.chom.2011.10.007, PMID: 22036470 PMC3227561

[ref74] Martens E. C. Chiang H. C. Gordon J. I. (2008). Mucosal glycan foraging enhances fitness and transmission of a saccharolytic human gut bacterial symbiont. Cell Host Microbe 4, 447–457. 10.1016/j.chom.2008.09.007, PMID: 18996345 PMC2605320

[ref75] Meng X. Wu H. Wang W. Lan T. Yang W. Yu D.. (2020). A purified aspartic protease from *Akkermansia muciniphila* plays an important role in degrading Muc2. Int. J. Mol. Sci. 21:72. 10.3390/ijms21010072, PMID: 31861919 PMC6982040

[ref76] Milani C. Turroni F. Duranti S. Lugli G. A. Mancabelli L. Ferrario C.. (2016). Genomics of the genus *Bifidobacterium* reveals species-specific adaptation to the glycan-rich gut environment. Appl. Environ. Microbiol. 82, 980–991. 10.1128/AEM.03500-15, PMID: 26590291 PMC4751850

[ref77] Morio A. Xu J. Masuda A. Kinoshita Y. Hino M. Morokuma D. (2019). Expression, purification, and characterization of highly active endo-α-*N*-acetylgalactosaminidases expressed by silkworm-baculovirus expression system. J. Asia Pac. Entomol. 22, 404–408. 10.1016/j.aspen.2019.01.009

[ref78] Neelima Sharma R. Rajput Y. S. Mann B. (2013). Chemical and functional properties of glycomacropeptide (GMP) and its role in the detection of cheese whey adulteration in milk: a review. Dairy Sci. Technol. 93, 21–43. 10.1007/s13594-012-0095-0, PMID: 23396893 PMC3567326

[ref79] Nishiyama K. Yamamoto Y. Sugiyama M. Takaki T. Urashima T. Fukiya S.. (2017). *Bifidobacterium bifidum* extracellular sialidase enhances adhesion to the mucosal surface and supports carbohydrate assimilation. mBio 8, e00928–e01117. 10.1128/mBio.00928-17, PMID: 28974612 PMC5626965

[ref80] O’Callaghan A. van Sinderen D. (2016). Bifidobacteria and their role as members of the human gut microbiota. Front. Microbiol. 7:925. 10.3389/fmicb.2016.00925, PMID: 27379055 PMC4908950

[ref81] O’Riordan N. Callaghan J. O. Buttò L. F. Kilcoyne M. Joshi L. Hickey R. M. (2018). Bovine glycomacropeptide promotes the growth of *Bifidobacterium longum* ssp. *infantis* and modulates its gene expression. J. Dairy Sci. 101, 6730–6741. 10.3168/jds.2018-14499, PMID: 29803426

[ref82] Ottman N. Geerlings S. Y. Aalvink S. de Vos W. M. Belzer C. (2017). Action and function of *Akkermansia muciniphila* in microbiome ecology, health and disease. Best Pract. Res. Clin. Gastroenterol. 31, 637–642. 10.1016/j.bpg.2017.10.001, PMID: 29566906

[ref83] Ouwerkerk J. P. van der Ark K. C. H. Davids M. Claassens N. J. Finestra T. R. de Vos W. M.. (2016). Adaptation of *Akkermansia muciniphila* to the oxic-anoxic interface of the mucus layer. Appl. Environ. Microbiol. 82, 6983–6993. 10.1128/AEM.01641-16, PMID: 27663027 PMC5103097

[ref84] Padra M. Adamczyk B. Benktander J. Flahou B. Skoog C. Padra J. T.. (2018). *Helicobacter suis* binding to carbohydrates on human and porcine gastric mucins and glycolipids occurs via two modes. Virulence 9, 898–918. 10.1080/21505594.2018.1460979, PMID: 29638186 PMC5955484

[ref85] Pinho S. S. Reis C. A. (2015). Glycosylation in cancer: mechanisms and clinical implications. Nat. Rev. Cancer 15, 540–555. 10.1038/nrc3982, PMID: 26289314

[ref86] Pluvinage B. Massel P. M. Burak K. Boraston A. B. (2019). Structural and functional analysis of four family 84 glycoside hydrolases from the opportunistic pathogen *Clostridium perfringens*. Glycobiology 30, 49–57. 10.1093/glycob/cwz069, PMID: 31701135 PMC6925667

[ref87] Png C. W. Lindén S. K. Gilshenan K. S. Zoetendal E. G. McSweeney C. S. Sly L. I.. (2010). Mucolytic bacteria with increased prevalence in IBD mucosa augment in vitro utilization of mucin by other bacteria. Am. J. Gastroenterol. 105, 2420–2428. 10.1038/ajg.2010.281, PMID: 20648002

[ref88] Podolsky D. K. (1985). Oligosaccharide structures of human colonic mucin. J. Biol. Chem. 260, 8262–8271. PMID: 4008490

[ref89] Praharaj A. B. Dehury B. Mahapatra N. Kar S. K. Behera S. K. (2018). Molecular dynamics insights into the structure, function, and substrate binding mechanism of mucin desulfating sulfatase of gut microbe *Bacteroides fragilis*. J. Cell. Biochem. 119, 3618–3631. 10.1002/jcb.26569, PMID: 29232003

[ref90] Puccio G. Alliet P. Cajozzo C. Janssens E. Corsello G. Sprenger N.. (2017). Effects of infant formula with human milk oligosaccharides on growth and morbidity. J. Pediatr. Gastroenterol. Nutr. 64, 624–631. 10.1097/MPG.0000000000001520, PMID: 28107288 PMC5378003

[ref91] Pudlo N. A. Urs K. Kumar S. S. German J. B. Mills D. A. Martens E. C. (2015). Symbiotic human gut bacteria with variable metabolic priorities for host mucosal glycans. mBio 6, e01282–e01315. 10.1128/mBio.01282-15, PMID: 26556271 PMC4659458

[ref92] Purcell R. V. Pearson J. Aitchison A. Dixon L. Frizelle F. A. Keenan J. I. (2017). Colonization with enterotoxigenic *Bacteroides fragilis* is associated with early-stage colorectal neoplasia. PLoS One 12:e0171602. 10.1371/journal.pone.0171602, PMID: 28151975 PMC5289627

[ref93] Quintana-Hayashi M. Padra M. Padra J. Benktander J. Lindén S. (2018). Mucus-pathogen interactions in the gastrointestinal tract of farmed animals. Microorganisms 6:55. 10.3390/microorganisms6020055, PMID: 29912166 PMC6027344

[ref94] Ravcheev D. A. Thiele I. (2017). Comparative genomic analysis of the human gut microbiome reveals a broad distribution of metabolic pathways for the degradation of host-synthetized mucin glycans and utilization of mucin-derived monosaccharides. Front. Genet. 8:111. 10.3389/fgene.2017.00111, PMID: 28912798 PMC5583593

[ref95] Ringot-Destrez B. Kalach N. Mihalache A. Gosset P. Michalski J. C. Léonard R.. (2017). How do they stick together? Bacterial adhesins implicated in the binding of bacteria to the human gastrointestinal mucins. Biochem. Soc. Trans. 45, 389–399. 10.1042/BST20160167, PMID: 28408479

[ref96] Rivière A. Selak M. Lantin D. Leroy F. De Vuyst L. (2016). Bifidobacteria and butyrate-producing colon bacteria: importance and strategies for their stimulation in the human gut. Front. Microbiol. 7:979. 10.3389/fmicb.2016.00979, PMID: 27446020 PMC4923077

[ref97] Robbe C. Capon C. Maes E. Rousset M. Zweibaum A. Zanetta J. -P.. (2003). Evidence of regio-specific glycosylation in human intestinal mucins. J. Biol. Chem. 278, 46337–46348. 10.1074/jbc.M302529200, PMID: 12952970

[ref98] Roberts G. Tarelli E. Homer K. A. Philpott-Howard J. Beighton D. (2000). Production of an endo-β-*N*-acetylglucosaminidase activity mediates growth of *Enterococcus faecalis* on a high-mannose-type glycoprotein. J. Bacteriol. 182, 882–890. 10.1128/JB.182.4.882-890.2000, PMID: 10648510 PMC94360

[ref99] Rojas E. Torres G. (2013). Isolation and recovery of glycomacropeptide from milk whey by means of thermal treatment. Food Sci. Technol. 33, 14–20. 10.1590/S0101-20612013005000027

[ref100] Ross S. A. Lane J. A. Kilcoyne M. Joshi L. Hickey R. M. (2015). “The milk fat globule membrane” in Biotechnology of bioactive compounds. eds. Gupta V. K. Tuohy M. G. O’Donovan A. Lohani M. (Chichester, UK: John Wiley & Sons, Ltd.), 631–668.

[ref101] Rossez Y. Maes E. Lefebvre Darroman T. Gosset P. Ecobichon C. Joncquel Chevalier Curt M.. (2012). Almost all human gastric mucin *O*-glycans harbor blood group a, B or H antigens and are potential binding sites for *Helicobacter pylori*. Glycobiology 22, 1193–1206. 10.1093/glycob/cws072, PMID: 22522599

[ref102] Saito T. Itoh T. (1992). Variations and distributions of *O*-glycosidically linked sugar chains in bovine κ-casein. J. Dairy Sci. 75, 1768–1774. 10.3168/jds.S0022-0302(92)77936-3, PMID: 1500573

[ref103] Sawicki C. Livingston K. Obin M. Roberts S. Chung M. McKeown N. (2017). Dietary fiber and the human gut microbiota: application of evidence mapping methodology. Nutrients 9:125. 10.3390/nu9020125, PMID: 28208609 PMC5331556

[ref104] Schroeder B. O. Birchenough G. M. H. Ståhlman M. Arike L. Johansson M. E. V. Hansson G. C.. (2018). Bifidobacteria or fiber protects against diet-induced microbiota-mediated colonic mucus deterioration. Cell Host Microbe 23, 27.e7–40.e7. 10.1016/j.chom.2017.11.004, PMID: 29276171 PMC5764785

[ref105] Schulz B. L. Sloane A. J. Robinson L. J. Prasad S. S. Lindner R. A. Robinson M.. (2007). Glycosylation of sputum mucins is altered in cystic fibrosis patients. Glycobiology 17, 698–712. 10.1093/glycob/cwm036, PMID: 17392389

[ref106] Seregin S. S. Golovchenko N. Schaf B. Chen J. Pudlo N. A. Mitchell J.. (2017). NLRP6 protects Il10−/− mice from colitis by limiting colonization of *Akkermansia muciniphila*. Cell Rep. 19, 733–745. 10.1016/j.celrep.2017.03.080, PMID: 28445725 PMC5528001

[ref107] Sheridan P. Martin J. C. Lawley T. D. Browne H. P. Harris H. M. B. Bernalier-Donadille A.. (2016). Polysaccharide utilization loci and nutritional specialization in a dominant group of butyrate-producing human colonic *Firmicutes*. Microb. Genom. 2:e000043. 10.1099/mgen.0.000043, PMID: 28348841 PMC5320581

[ref108] Shimada Y. Watanabe Y. Wakinaka T. Funeno Y. Kubota M. Chaiwangsri T.. (2015). α-*N*-acetylglucosaminidase from *Bifidobacterium bifidum* specifically hydrolyzes α-linked *N*-acetylglucosamine at nonreducing terminus of *O*-glycan on gastric mucin. Appl. Microbiol. Biotechnol. 99, 3941–3948. 10.1007/s00253-014-6201-x, PMID: 25381911

[ref109] Shin J. Noh J. R. Chang D. H. Kim Y. H. Kim M. H. Lee E. S.. (2019). Elucidation of *Akkermansia muciniphila* probiotic traits driven by mucin depletion. Front. Microbiol. 10:1137. 10.3389/fmicb.2019.01137, PMID: 31178843 PMC6538878

[ref110] Shokryazdan P. Faseleh Jahromi M. Navidshad B. Liang J. B. (2017). Effects of prebiotics on immune system and cytokine expression. Med. Microbiol. Immunol. 206, 1–9. 10.1007/s00430-016-0481-y, PMID: 27704207

[ref111] Sicard J. -F. Le Bihan G. Vogeleer P. Jacques M. Harel J. (2017). Interactions of intestinal bacteria with components of the intestinal mucus. Front. Cell. Infect. Microbiol. 7:387. 10.3389/fcimb.2017.00387, PMID: 28929087 PMC5591952

[ref112] Stanley P. Taniguchi N. Aebi M. (2017). “N-glycans” in Essentials of glycobiology. eds. Varki A. Cummings R. D. Esko J. D. Stanley P. Hart G. W. Aebi M. (NY: Cold Spring Harbor Laboratory Press). 20301239

[ref113] Sunds A. V. Poulsen N. A. Larsen L. B. (2019). Short communication: application of proteomics for characterization of caseinomacropeptide isoforms before and after desialidation. J. Dairy Sci. 102, 8696–8703. 10.3168/jds.2019-16617, PMID: 31351722

[ref114] Tailford L. E. Crost E. H. Kavanaugh D. Juge N. (2015). Mucin glycan foraging in the human gut microbiome. Front. Genet. 6:81. 10.3389/fgene.2015.00081, PMID: 25852737 PMC4365749

[ref115] Thomä-Worringer C. Sørensen J. López-Fandiño R. (2006). Health effects and technological features of caseinomacropeptide. Int. Dairy J. 16, 1324–1333. 10.1016/j.idairyj.2006.06.012

[ref116] Thompson P. Medina D. A. Garrido D. (2018). Human milk oligosaccharides and infant gut bifidobacteria: molecular strategies for their utilization. Food Microbiol. 75, 37–46. 10.1016/j.fm.2017.09.001, PMID: 30056961

[ref117] Turroni F. Bottacini F. Foroni E. Mulder I. Jae-Han K. Zomer A.. (2010). Genome analysis of *Bifidobacterium bifidum* PRL2010 reveals metabolic pathways for host-derived glycan foraging. Proc. Natl. Acad. Sci. U. S. A. 107, 19514–19519. 10.1073/pnas.1011100107, PMID: 20974960 PMC2984195

[ref118] Turroni F. Milani C. Duranti S. Mahony J. van Sinderen D. Ventura M. (2018). Glycan utilization and cross-feeding activities by bifidobacteria. Trends Microbiol. 26, 339–350. 10.1016/j.tim.2017.10.001, PMID: 29089173

[ref122] Vandeputte D. Kathagen G. D’hoe K. Vieira-Silva S. Valles-Colomer M. Sabino J.. (2017). Quantitative microbiome profiling links gut community variation to microbial load. Nature 551, 507–511. 10.1038/nature24460, PMID: 29143816

[ref119] van der Ark K. C. H. Aalvink S. Suarez-Diez M. Schaap P. J. de Vos W. M. Belzer C. (2018). Model-driven design of a minimal medium for *Akkermansia muciniphila* confirms mucus adaptation. Microb. Biotechnol. 11, 476–485. 10.1111/1751-7915.13033, PMID: 29377524 PMC5902328

[ref120] van Passel M. W. J. Kant R. Zoetendal E. G. Plugge C. M. Derrien M. Malfatti S. A.. (2011). The genome of *Akkermansia muciniphila*, a dedicated intestinal mucin degrader, and its use in exploring intestinal metagenomes. PLoS One 6:e16876. 10.1371/journal.pone.0016876, PMID: 21390229 PMC3048395

[ref121] van Tassell M. L. Miller M. J. (2011). *Lactobacillus* adhesion to mucus. Nutrients 3, 613–636. 10.3390/nu3050613, PMID: 22254114 PMC3257693

[ref123] Varki A. Lowe J. B. (2009). “Biological roles of glycans” in Essentials of glycobiology. eds. Varki A. Cummings R. Esko J. Freeze H. Hart G. Marth J. (NY: Cold Spring Harbor Laboratory Press). 20301233

[ref124] Vliegenthart J. F. (2017). The complexity of glycoprotein-derived glycans. Proc. Jpn. Acad. Ser. B Phys. Biol. Sci. 93, 64–86. 10.2183/pjab.93.005, PMID: 28190870 PMC5422628

[ref125] Wang M. Zhang X. Y. Guo R. R. Cai Z. P. Hu X. C. Chen H.. (2018). Cloning, purification and biochemical characterization of two β-*N*-acetylhexosaminidases from the mucin-degrading gut bacterium *Akkermansia muciniphila*. Carbohydr. Res. 457, 1–7. 10.1016/j.carres.2017.12.007, PMID: 29304441

[ref126] Wexler A. G. Goodman A. L. (2017). An insider’s perspective: bacteroides as a window into the microbiome. Nat. Microbiol. 2:17026. 10.1038/nmicrobiol.2017.26, PMID: 28440278 PMC5679392

[ref127] Wilson B. Whelan K. (2017). Prebiotic inulin-type fructans and galacto-oligosaccharides: definition, specificity, function, and application in gastrointestinal disorders. J. Gastroenterol. Hepatol. 32, 64–68. 10.1111/jgh.13700, PMID: 28244671

[ref128] Wrzosek L. Miquel S. Noordine M. L. Bouet S. Chevalier-Curt M. J. Robert V.. (2013). *Bacteroides thetaiotaomicron* and *Faecalibacterium prausnitzii* influence the production of mucus glycans and the development of goblet cells in the colonic epithelium of a gnotobiotic model rodent. BMC Biol. 11:61. 10.1186/1741-7007-11-61, PMID: 23692866 PMC3673873

[ref129] Yamada T. Hino S. Iijima H. Genda T. Aoki R. Nagata R.. (2019). Mucin *O*-glycans facilitate symbiosynthesis to maintain gut immune homeostasis. EBioMedicine 48, 513–525. 10.1016/j.ebiom.2019.09.008, PMID: 31521614 PMC6838389

[ref130] Yamamoto T. Ugai H. Nakayama-Imaohji H. Tada A. Elahi M. Houchi H.. (2018). Characterization of a recombinant *Bacteroides fragilis* sialidase expressed in *Escherichia coli*. Anaerobe 50, 69–75. 10.1016/j.anaerobe.2018.02.003, PMID: 29432848

[ref131] Yassour M. Jason E. Hogstrom L. J. Arthur T. D. Tripathi S. Siljander H.. (2018). Strain-level analysis of mother-to-child bacterial transmission during the first few months of life. Cell Host Microbe 24, 146.e4–154.e4. 10.1016/j.chom.2018.06.007, PMID: 30001517 PMC6091882

[ref132] Zhang T. Yang Y. Liang Y. Jiao X. Zhao C. (2018). Beneficial effect of intestinal fermentation of natural polysaccharides. Nutrients 10:1055. 10.3390/nu10081055, PMID: 30096921 PMC6116026

[ref133] Zúñiga M. Monedero V. Yebra M. J. (2018). Utilization of host-derived glycans by intestinal *Lactobacillus* and *Bifidobacterium* species. Front. Microbiol. 9:1917. 10.3389/fmicb.2018.01917, PMID: 30177920 PMC6109692

